# Factors Associated with Food Insecurity Following Hurricane Harvey in Texas

**DOI:** 10.3390/ijerph17030762

**Published:** 2020-01-25

**Authors:** Lauren A. Clay, Ashley D. Ross

**Affiliations:** 1 Health Administration and Public Health Department, D’Youville College, Buffalo, NY 14201, USA; 2 College of Global Public Health, New York University, New York, NY 10003, USA; 3 Marine Sciences Department, Texas A&M University at Galveston, Galveston, TX 77550, USA; ashleydross@tamug.edu

**Keywords:** food security, disaster, hurricane, Hurricane Harvey

## Abstract

Food insecurity prevalence among disaster-affected households has been found to be higher than state prevalence in non-disaster times. This study applies a socio-ecological model of post-disaster food insecurity to a nested quota sample (*n* = 1002) recruited for a web survey from 41 Texas counties affected by Hurricane Harvey 12–15 months post-event. This analysis identifies risk and protective factors for food insecurity. Chi-square analysis was used to examine independent associations between individual, household, and social factors with food insecurity. A multivariate logistic model was fitted and adjusted odds ratios are reported. Economic instability (adjusted odds ratio (OR) 2.43; 95% Confidence Interval (CI) 1.73, 3.41), relocation due to Hurricane Harvey (OR 1.89; CI 1.15, 3.09), major home damage (OR 2.11; CI 1.12, 3.98), non-white race – black (OR 1.79; CI 1.01, 3.18), Hispanic (OR 1.67; CI 1.09, 2.54), other race (OR 4.39; CI 1.96, 9.82) – and community-based organization assistance (1.99; 1.11, 3.58) were risk factors while older age (45–64 years: 0.49; 0.32, 0.73; 65+ years 0.40; 0.22, 0.75), better physical health (0.46; 0.29, 0.71), better mental health (0.46; 0.32, 0.67), and high social support (0.37; 0.25, 0.55) were protective against food insecurity. Disaster policies and programs should address the disproportionate burden on households that relocate or have health conditions. Fostering social support networks, especially among relocated populations, may improve disaster health outcomes.

## 1. Introduction

The United States Department of Agriculture (USDA) defines low food security as “reduced quality, variety, or desirability of diet” with “little or no indication of reduced food intake” and very low food security as “multiple indications of disrupted eating patterns and reduced food intake” [1]. Food insecurity poses serious health risks such as poor nutrition [2,3], cardiovascular disease [4], poor quality of life [5], poor self-rated physical and mental health [6], and poor functional health and restricted activity [7]. Food insecurity has also been associated with poor health outcomes in children, including higher rates of acute infections, developmental problems, mental health problems [8], and chronic conditions [3,9,10,11,12]. Food insecurity disproportionately affects households in rural areas, headed by a single adults with children [13], predominantly Black (non-Hispanic) households [14], and households with children under the age of six—many of the same groups that are socially vulnerable to disasters [15]. Disasters also increase risk for food insecurity as prevalence is higher among individuals who experience a change in their life circumstances such as changes in financial obligations, household composition, or housing stability [13,16], all possible consequences of disasters for exposed households.

Disasters cause pervasive disruption across all levels of the socio-ecological model. Individuals experience stress potentially due to witnessing the disaster, sheltering or evacuating, displacement, or disruption to normal routines [17,18,19,20,21]. Households may experience damage to their home, stress on familial relationships when coping with disaster impacts, or changes in household material or financial resources as a result of disaster exposure [22,23,24]. At the social group level, friend, neighbor, or co-worker relationships may be impacted through the sharing of support and resources and changes in social networks may occur due to displacement [25,26,27]. Finally, at the community level, disruption to normal community functioning from disaster-damaged infrastructure is common such as temporary school closures and reduction in the availability of critical lifelines such as water or electricity services [28,29]. Impacts at each of these levels may also impact food security. For example, disruption to food supply chains, closure of damaged food stores, or depleted financial or kitchen resources may impact an individual’s ability to access sufficient food or the availability of acceptable food to meet food needs [13,16,30].

A Proquest literature search conducted in December 2019 for the search terms “food insecurity AND disaster AND U.S.” returned 1871 peer reviewed articles. A close review of the 100 most relevant articles in that search found that 54 articles were not focused on the United States (US), 36 articles focused on food or disasters but not both, four discussed food and disasters but were not food insecurity focused rather focused on aspects such as agricultural practices or disaster preparedness, one article was a historical piece on 17th century food insecurity [31], and one manuscript was a systematic literature review of the disaster literature [32]. The systematic review found that less than one percent of disaster research focuses on food [32], supporting our literature review finding of only three studies that directly address food insecurity outcomes following disasters in the US. These three studies indicate that food insecurity prevalence is higher than state level prevalence among households impacted by disasters and that disasters such as hurricanes have widespread impacts on the local food environment from production to consumption [33,34,35,36]. These studies also demonstrate that food security is a post-disaster challenge for affected individuals and households and warrants additional investigation to understand the risk and protective factors for food security outcomes so that public health and emergency management programs and policies can more effectively mitigate adverse health outcomes of disasters. To further advance our understanding of the causal drivers of food insecurity following disaster exposure in the US, risk and protective factors should be examined in different hazard events, for different hazard types, and with different sampling frames to establish evidence for consistency, association, and specificity [37]. The present study does this and, therefore, will provide further empirical data on the magnitude of challenges of post-disaster food insecurity. 

Hurricane Harvey and the experience of Texas residents offers an opportunity to examine factors associated with food insecurity in a different hazard event and using a different sampling frame to further characterize how disasters may impact food security in affected areas. The present analysis examines risk and protective factors of post-disaster food insecurity among Texas households in counties with a federal disaster declaration for Hurricane Harvey across the socio-ecological model. Socio-ecological theory posits that health outcomes and behaviors are influenced by complex and interacting factors across multiple levels of influence including the individual, social (family or social group), community, institutional, and policy levels [38,39]. Socioecological theory has been used in studies of food insecurity and it is the primary theoretical orientation of the Dietary Guidelines for Americans 2015–2020 [40,41,42]. From an ecological perspective, the health outcome of food insecurity is influenced by factors from across the socioecological model. Individual level factors such as physical and mental health have been associated with food insecurity [34]; a person’s race and ethnicity, income, and partnership can also influence such issues [14,43,44,45,46]. At the household or social level, housing stability, social support, coping, and family decision making under financial constraint influence food security [47,48,49]. At the community and environmental levels, community cohesion, built environment features such as location of food stores and transportation networks, and local food sources influence food access [50,51,52]. At the institutional level, food safety net programs, changes in those services post-disaster, and civic and social institutional roles in food provision influence food security [53,54,55,56,57]. Finally, at the policy and societal level, the allocation of resources as well as social, economic, and political forces influence food insecurity outcomes for communities [58,59]. The present analysis applies a socioecological framework to examine factors at the individual, household, and social levels of influence following Hurricane Harvey in Texas.

**Figure 1:**
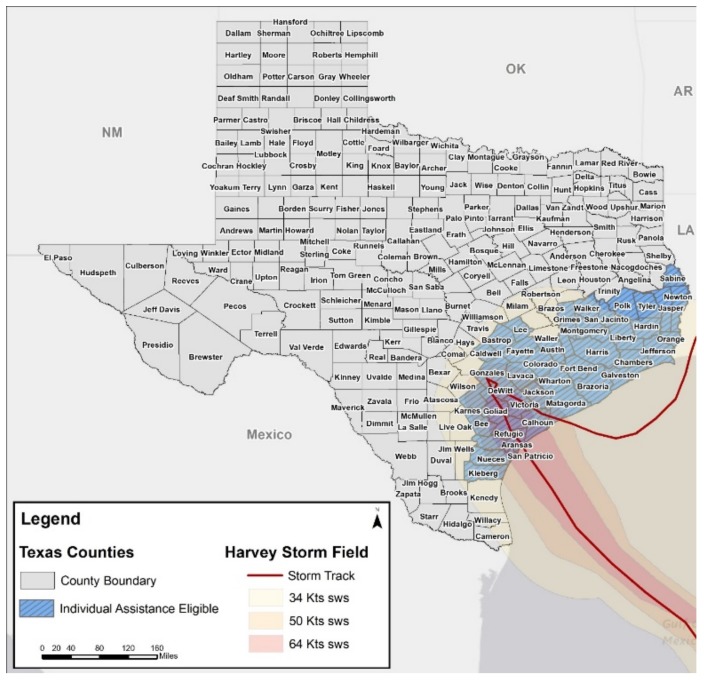
Hurricane Harvey storm track across Texas.

Note: Hurricane Harvey storm fields are illustrated using the National Hurricane Center’s best track estimate and maximum sustained wind swaths (knots) of the storm. Shaded counties are those eligible for Federal Emergency Management Agency (FEMA) Individual Assistance.

Hurricane Harvey made landfall around 22:00 on 25 August 2017 in Rockport, Texas. It was the first major hurricane to affect this area since Hurricane Celia in 1970 [60]. Peak wind gusts were recorded at 132 mph, and storm surge levels reached more than 12 feet above ground level in some areas [61]. Rather than the typical movement inland and away from the coast, Harvey stalled over Southeast Texas for days causing torrential rain and flash flooding [62] (Figure 1). Over the week that Harvey hammered the state, rainfall totals peaked at 60.58 inches across Texas [61], exceeding average annual rainfall totals for the area [63] and making it the most significant tropical cyclone rainfall event in US history [64]. The excessive precipitation Harvey dumped on Texas resulted in reservoir and riverine flooding throughout the state [65]. Harvey caused $125 billion in damages, making it the second most costly disaster in US history behind Hurricane Katrina’s $161 billion in damages [66]. The present analysis aims to identify factors at the individual, household, and social group levels associated with individual food insecurity in the aftermath of Hurricane Harvey in Texas. We hypothesize that factors across all levels of the socio-ecological model will be associated with food insecurity.

## 2. Materials and Methods

### 2.1. Sample

In the state of Texas, 49 counties were eligible for Federal Emergency Management Agency (FEMA) Community Development Block Grant—Disaster Recovery assistance; these counties comprise a land area roughly the size of the state of Kentucky [67]. Within this large affected area, there is a diverse set of communities with varying social, economic, and environmental characteristics. This study leverages the diversity of the affected area to examine resilience in rural communities; it is a part of a larger mixed-methods study, funded by the National Academies of Science, Engineering, and Medicine (NASEM) Gulf Research Program, to assess the experiences of households affected by Harvey with a particular focus on rural communities [68]. A sample of 1002 individuals were recruited to complete a cross-sectional online survey from the population of interest: the 41 Texas counties eligible for FEMA Individual Assistance following Hurricane Harvey. The panel was filled by Qualtrics to meet a set of nested quotas on selected demographic characteristics for two groups—urban and rural. To make the two groups of the sample representative, the quotas were matched to population proportions for the respective group on age, sex, and race and ethnicity, determined by US Census Bureau data [69]. To designate a county as rural or urban, we compared the Texas Health and Human Services (HHSC) designations to the definitions of rural in Texas statutes and the Texas Administrative Code [70]. Where there was disagreement between the two sources, we used the more detailed information provided by the USDA Economic Research Service rural-urban commuting area codes [71]. These codes classify census tracts using measures of population density, urbanization, and daily commuting. In cases of designation conflict between Texas HHSC and the state of Texas, we define a county as urban if the majority of the county population resides in a metropolitan area. Based on this designation schema, 22 of the counties are considered rural while 19 are coded urban; of the 1002 respondents, 487 resided in rural areas (48.6%) and 515 resided in urban areas (51.4%). Rural respondents were purposively oversampled—with regards to total population size—so that there would be a sufficient number of observations to analyze in comparison to the urban group. In terms of quotas, the urban sample closely matched population characteristics, noted in parentheses, for: age—12.62% (12.80%) 18–24 years old, 33.98% (33.90%) 25–44 years old, 36.12% (35.50%) 45–64 years old, and 17.28% (17.80%) 65+ years; sex—47.96% (49.30%) male and 52.04% (50.70%) female; and race/ethnicity—57.28% (57.00%) white, 10.87% (11.10%) black, 26.02% (27.80%) Hispanic or Latino, and 5.63% (4.10%) other. Race and ethnicity were closely matched in the rural sample to population parameters, again reported in parentheses: 57.29% (59.1%) white, 9.24% (9.80%) black, 28.95% (29.00%) Hispanic or Latino, and 4.52% (2.10%) other. However, due to limited availability of rural respondents, it was not possible to match age and sex as closely in the rural sample: age—19.51% (12.2%) 18–24 years old, 36.34% (30.10%) 25–44 years old, 31.01% (35.20%) 45–64 years old, and 13.14% (22.50%) 65+ years; sex—36.96% (51.80%) male and 63.04% (48.20%) female. Given these discrepancies between the quotas and population proportions, a sample weight was calculated to adjust the sample to population parameters for age, sex, and race/ethnicity using a “raking” or iterative proportional fitting method [72]. While applying the weight to the quota-based sample adjusts the sample to make it representative of the population, there are unknown biases introduced into the survey estimates. This is due to the non-probability sampling frame because measures of precision (i.e., response rate, margins of error) are not available with such a sampling approach. Study exclusionary criteria were being under age 18 and residing outside the 41 Hurricane Harvey federally disaster declared counties in Texas eligible for FEMA Individual Assistance Programs.

### 2.2. Data Collection Procedures

The online survey is the second phase of data collection of the larger study. The survey instrument was built upon themes emergent from 108 semi-structured qualitative interviews conducted in the first phase of this study. The first phase of data collection took place 2–3 months following Hurricane Harvey and recruited individuals from four affected communities in Texas, including three rural cases and one urban case [68]. The online survey included 80 questions that asked respondents about recovery experiences from Hurricane Harvey, perceptions of their community, disaster preparedness, personal health, and home and household characteristics. The survey was in the field from 24 October–4 December 2018. Given the focus of portions of the questionnaire (i.e., assistance, social support) on disaster recovery, and the relatively long timeline for recall of disaster experiences established in the disaster literature [73], data collection approximately a year after the event is appropriate. The Texas A&M University Institutional Review Board approved this study (reference number 096747).

### 2.3. Measures

The measures selected capture individual data from across the individual, household, and social group levels of the socio-ecological model identified in the disaster and food security literature as important for understanding disaster experience and health. The outcome food security was assessed with a validated two-item food security screener (97 percent sensitivity, 83 percent specificity) that was designed to rapidly identify individuals at-risk for food insecurity [74]. Participants were asked to report how often [often true, sometimes true, never true] since Hurricane Harvey the following two statements were true for their family: “we have worried whether our food would run out before we got money to buy more” and “the food we bought just didn’t last and we didn’t have money to get more.” Consistent with other studies of food insecurity [75,76,77,78], respondents indicating “often true” or “sometimes true” for either measure were categorized as at-risk for food insecurity while respondents indicating “never true” for either question were categorized as food secure.

At the individual level, physical and mental health were assessed with the SF-12, a validated and standardized measure of physical health and mental health. The SF-12 was developed from the SF-36 Health Survey, the most widely used health metric in the world [79]. The SF-12 has been used to measures mental and physical health worldwide in studies of a broad range of health outcomes [79]. It has been widely used in disaster studies to measure physical and mental health [34,80,81,82,83]. The mental health (MCS) and physical health (PCS) component scores are norm-based and were computed by transforming linear scores to achieve a mean of 50 and standard deviation of 10 in the US general population with QualityMetric scoring software (Optum, Johnston, RI, USA) [84,85]. Normative data enable the comparison of individuals or groups by comparing them with the distribution of scores for other individuals. Therefore, scores are not absolute but represent the departure from the typical score [79]. Respondents in this sample were categorized as well below, below, and the same or better as the general population [85]. The benefits of using normed scores for the SF-12 are that the MCS and PCS scores can be directly and meaningfully interpreted across scales. Individual level demographic characteristics included age [reported in years], race [white, Hispanic or Latino, black or African-American, Asian, American-Indian or Alaska native, Native Hawaiian or Pacific Islander, Middle Eastern or North African, other; categorized as white, Hispanic, black, and other], sex [male, female], education [high school, some college, Associate’s degree, technical degree or certification, Bachelor’s degree, Master’s degree, Doctoral degree, Medical degree, and Law degree; categorized as high school, some college, college degree, and graduate degree] and employment status [self-employed, work full-time for an employer or the military, work part-time for an employer or the military, homemaker, full-time student, permanently sick, disabled, or unable to work, unemployed or temporarily laid off, retired; dichotomized as employed and unemployed].

At the household level, income was self-reported by respondents for the household including wages, tips, investment income, public assistance, and income from retirement plans [less than $15,000; $15,000–24,999; $25,000–34,999; $35,000–49,999; $50,000–74,999; $75,000–99,999; $100,000–149,999; $150,000 or more]. Economic stability was assessed by asking respondents whether they lost a job or lost income as a result of Hurricane Harvey. Responses were dichotomously coded, and respondents were classified as having economic instability if they reported loss of job or income due to Hurricane Harvey. Home damage was assessed by asking respondents to describe the impact of Hurricane Harvey on their home using the FEMA Housing Damage Assessment categories and descriptions of not damaged, affected (some damage to the structure and contents, but still could be lived in), minor damage (home is damaged and could not be lived in, but was livable again within a short period of time—approximated 30 days), major damage (substantial failure to structural elements of residence, e.g., walls, floors, foundation, or damage that required more than 30 days to repaid, the home could not be lived in during that time), or destroyed (total loss of structure, structure not economically feasible to repair). Responses were dichotomized as major damage where the household was displaced for a period of time (major damage, destroyed) and little to no damage for all other categories (not damaged, affected, minor damage) [86]. Relocation was assessed by asking study participants if their household moved out of their community due to Hurricane Harvey (yes/no). For this survey question, community was not defined geographically but rather referred to what the respondent considers their own community to be. Rural was assigned by county of residence as described in the sampling frame description above, and homeownership was assessed by asking whether participants currently rent or own their home (rent/own).

At the social group level, social support was assessed by asking participants if they have someone they can count on for five types of support (to help with everyday favors, to take care of you if confined to a bed for several weeks, to lend you several hundred dollars for a medical emergency, to talk to if you were having trouble with a family relationship, to help you find local housing if you had to move) [35,87]. Respondents indicating support for two or more types of support were categorized as having high social support while those reporting fewer than two types of support were categorized as having low or no social support. Assistance following Hurricane Harvey was assessed by asking survey respondents if they received any financial assistance for recovery from any of the following sources: FEMA, Small Business Association, American Red Cross, own savings or assets, family, church or faith-based organizations, non-profit organizations, bank or financial institutions, neighborhood organization, crowdsourced funding like Go Fund Me, or other sources. Respondents indicating assistance from faith-based, non-profits, and neighborhood organizations were categorized as having community-based organization assistance.

### 2.4. Data Analysis

Using a model-building approach, each exposure variable was examined independently with a chi-square analysis for association with the outcome: post-disaster food insecurity. All factors that demonstrated an independent statistically significant association with the outcome were retained for multivariate analysis. A series of three logistic regression models were fitted with the survey weight to adjust the sample to population parameters on age, sex, and race/ethnicity, which is an appropriate approach for the quota-based, non-probability study sample [88]. Logistic regression, widely used in epidemiological and social science research, was chosen as the method of analysis because it has been shown to be less restrictive than linear analysis for modeling categorical outcomes, more efficient for analyzing dichotomous dependent variables, and the most consistent in estimating parameters regardless of the distribution of the independent variables [89,90]. Model one included the individual level factors significantly associated with the outcome, including age, race, education, physical health, and mental health. The second model retained all individual level variables significantly associated with the outcome and added household level variables including income, economic stability, homeowner, home damage, and relocation due to Harvey. The final model retained all variables associated with the outcome from model two and added social level measures of social support and assistance from a community-based organization. The log-likelihood, Hosmer-Lemeshow goodness of fit, Akaike information criterion, and Bayesian information criterion tests were performed to determine the best fitting model [91,92,93]. Multivariate regression results were evaluated for statistical significance at the p ≤ 0.05 level. Adjusted odds ratios and 95% confidence intervals are reported to evaluate the associations between individual, household, and social level factors with the outcome. Stata version 16 (StataCorp, College Station, TX, USA) was used to conduct the statistical analyses [94].

## 3. Results

### 3.1. Sample Description

Sample characteristics are reported in Table 1. Approximately half of study participants reported age 18–44 years. More than half the sample was white (57.3 percent). Just over 10 percent of the sample reported being unemployed (10.6 percent). Some college was the education category most selected by study participants (42.7 percent). Nearly three-quarters of our sample reported physical health that is better than the general population (71.5 percent), and over half (57.2 percent) of the sample reported mental health better than the general population.

At the household level, between 6.3 percent and 20.0 percent of respondents were in each income level with the greatest number of respondents falling in the $35,000–49,999 range. About half of study participants reported loss of a job or income due to Hurricane Harvey (52.7 percent), and 62.4 percent reported owning their homes. Just over 10 percent of the sample reported major home damage, and 16.5 percent reported relocation due to Hurricane Harvey. As previously detailed, 48.6% of the sample reside in rural areas while 51.4% reside in urban areas. At the social group level, three-quarters of the sample report high social support (76.4 percent), and nine percent of respondents reported receiving assistance from a community-based organization after Hurricane Harvey.

### 3.2. Bivariate Analysis

Examination of individual level characteristics of study participants showed that age, race, education, unemployment, physical health, and mental health had a statistically significant association with the outcome food insecurity after Hurricane Harvey. Table 2 reports the factors that exhibit statistically significant independent associations (χ^2^) with the outcome as well as the incidence of each factor among the subgroups of food secure and food insecure. At the household level, income, economic stability, home ownership, level of home damage, and relocation due to Harvey were significantly associated with the outcome. Finally, at the social level, both social support and whether the household received assistance from a community-based organization following Harvey were significantly associated with the outcome.

### 3.3. Multivariate Analysis

When examining the individual level factors associated with the outcome food insecurity in an logistic regression model (model 1), older age, college degree, better physical health, and better mental health were protective against food insecurity while non-white race was a risk factor (Table 3). Study participants age 45–64 years old were 47 percent less likely to report food insecurity than respondents age 18–44 years old (adjusted odds ratio (OR) 0.53; 95% Confidence Interval (CI) 0.37, 0.76), and study participants age 65 years or older were 67 percent less likely than respondents age 18–44 years old to report food insecurity following Hurricane Harvey (OR 0.33; CI 0.19, 0.59). Respondents reporting a college degree were 40 percent less likely to report food insecurity than those with less than a high school education (OR 0.60; CI 0.39, 0.94).

Black participants were nearly two and half times more likely to report food insecurity than white participants (OR 2.42; CI 1.48, 3.98). Hispanic respondents had nearly twice the odds of reporting food insecurity compared to white respondents (OR 1.80; CI 1.24, 2.61), and respondents identifying with “other” race had nearly four times the odds of reporting food insecurity (OR 3.85; CI 1.91, 7.75). Individuals reporting physical health the same or better than the general population were 69 percent less likely to report food insecurity compared to individuals reporting physical health well below the general population (OR 0.31; CI 0.21, 0.46). Similarly, individuals reporting mental health the same or better than the general population were 71 percent less likely to report food insecurity (OR 0.29; CI 0.21, 0.40), and individuals reporting mental health below the general population were 38 percent less likely to report food insecurity (OR 0.63; CI 0.40, 1.00) than individuals reporting mental health well below the general population.

Model two added household level factors that were significantly associated with the outcome food insecurity. All associations from model one remained statistically significant with the exception of education and unemployment, which were dropped after post-testing revealed they did not strengthen the model. Among the household level variables, higher income was protective, and economic instability, major home damage, and relocation due to Harvey were risk factors for food insecurity. Greater income was found to be protective against post-Harvey food insecurity for all income ranges compared to the category of less than $15,000. Respondents reporting economic instability, measured as loss of a job or income due to Harvey, had 2.42 times the odds of reporting food insecurity compared to those that did not report economic losses due to Harvey (OR 2.42; CI 1.74, 3.38). Study participants reporting major home damage in Hurricane Harvey had 2.41 greater odds of reporting food insecurity following the storm compared to those with little to no damage (OR 2.41; CI 1.28, 4.55). Participants reporting relocation due to Harvey had nearly double the odds of reporting food insecurity following Harvey (OR 1.86; CI 1.15, 3.02) compared to those this did not report relocation.

Model three added social level factors to the model. Mental health below the general population lost statistical significance while mental health reported as the same or better than the general population remains a statistically significant protective factor (OR 0.46; CI 0.32, 0.67). Lower income ranges also lose statistical significance when included in the model alongside social factors, but higher incomes remained protective and statistically significant. In post-testing for model two, home ownership did not strengthen the model and, therefore, was not retained for model three. The remaining individual and household level factors persist as statistically significant predictors of post-Harvey food insecurity. When looking at the social factors, social support was protective against food insecurity (OR 0.37; CI 0.25, 0.55) while assistance from community-based organizations was a risk factor (OR 1.99; CI 1.11, 3.58).

## 4. Discussion

In this study of households residing in the 41 Texas counties impacted by Hurricane Harvey (measured by eligibility for FEMA Individual Assistance Programs), the prevalence of post-disaster food insecurity was high. Accounting for factors at the individual, household, and social level, we found that minority (non-white) race, economic instability, major home damage, relocation due to Harvey, and receiving assistance from community-based organizations were risk factors for food insecurity. Older age, better physical health, better mental health, greater income, and high social support were protective factors against food insecurity.

We hypothesized that factors at the individual, household or family, and social group level of the socio-ecological model would be associated with the outcome food insecurity. Our hypothesis was supported by the results of multiple statistical analyses. At the individual level, race, age, physical health, and mental health were associated with food insecurity outcomes. At the household or family level, economic stability, home damage, relocation, and income were associated with food insecurity. Finally, at the social group level, social support and assistance from community organizations were associated with food insecurity. 

The food insecurity literature demonstrates that rural households are more at risk for food insecurity and that disruption to life circumstances, such as housing instability and change in financial obligations, increase the likelihood of food insecurity [13,15,16]. In the present analysis, living in a rural designated county did not exhibit an independent, statistically significant association with food insecurity. The study design oversampled rural households to capture the rural experience with disaster as part of a larger study of resilience. From this design, we expected a greater incidence of food insecurity among rural participants in our sample based on the food insecurity literature. This finding may imply that rural households are better able to mobilize their social networks for resources in a landscape that includes fewer formal resources and assistance following disasters; there is some evidence of this following Hurricane Harvey [68,95]. However, more study of this is needed to support such conclusions. Additional research exploring the experience of rural households as it relates to meeting basic needs following disasters will shed more light on the potential mechanisms for meeting household food and other needs following disaster exposure.

Past research examining a cohort of displaced households following Hurricane Katrina found that higher income, having a partner, and high social support were protective, while “other” race (not white, Black, or Hispanic), poor mental health, poor physical health, and female sex were risk factors for food insecurity [34,35]. The present analysis, examining a different sample (a quota-based sample of households from 41 Texas counties eligible for FEMA Individual Assistance following Hurricane Harvey), found similar risk and protective factors while accounting for displacement. Higher income and social support, better physical health, and better mental health were protective and “other” race were risk factors; however, female sex was not significantly associated with food insecurity outcomes. In addition to “other” race, after adjusting for individual, household, and social factors, black and Hispanic race/ethnicity were also risk factors for food insecurity following Harvey. Another notable finding of the Katrina cohort study is that housing stability, measured by number of moves since Hurricane Katrina and number of years to achieving stable housing, was not associated with food security outcomes [34,35]. This study, of a different disaster event, in a different geographic context, and with a different sampling frame, departs from that finding but aligns with the food security literature indicating housing instability is a risk factor for food insecurity. In the present analysis, level of home damage and relocation were both risk factors for food insecurity following Harvey. Additional analysis of study participants that were displaced, indicated by relocation or home destroyed, is needed to further explore this finding.

Assistance from a community-based organization was identified as a risk factor for food insecurity in the present analysis. Due to the cross-sectional nature of this study, we are not able to determine whether the risk is related to a need for assistance from community organizations or whether those who may experience times of food insecurity are already receiving services or assistance from community organizations. Additional research on the role of community organizations in meeting community food needs following disasters and longitudinal research that can capture temporal relationships are important to better understand this association.

The current study is limited by its cross-sectional design. Such an approach captures exposure and outcome measures at the same time, but a temporal, causal relationship cannot be established. The strengths of this approach for examining factors associated with food insecurity following disaster exposure outweigh this limitation as this is a new area of research with few empirical studies examining post-disaster food security outcomes. The present cross-sectional analysis has provided additional information on the experience of individuals in disaster-affected communities with food security. This information advances our understanding of the prevalence of food insecurity following disasters and the potential factors contributing to those outcomes – critical information to move forward with further research in this area. The study is also limited by its reliance on online survey data where participants were recruited. This approach samples only individuals with access to online services. Furthermore, to fill the quota-based panel Qualtrics invited individuals to participate via online mechanisms (i.e., banners, emails). Therefore, study findings should be interpreted with an understanding that online, opt-in surveys, such as the one used in this study, attract more politically and civically engaged individuals [96]. This may bias the study results as such individuals, with presumably greater social capital, may have better access to social resources that facilitate disaster recovery [97,98]. 

Another limitation of the present study is that the quota-based survey sample relies on a non-probability sampling frame. Increasingly, non-probability surveys are being used by researchers due to low response rates, high costs, and poor coverage of probability surveys [88]. While quota-based sampling aims to match a panel to a set of population parameters and, therefore, increase the representativeness of the sample, non-probability samples do not allow for calculation of margins of error that provide a measure of precision. This results in introducing unknown sampling biases into the survey estimates [99]. A study by Pew Research Center concludes that such biases may be reduced through the use of survey weights [96]. Accordingly, this study includes a weight that adjusts the sample on population parameters for sex, race/ethnicity, and age using an iterative proportional fitting method by Bergmann [72]. This method is appropriate for managing the limitations of non-probability survey samples [88] but does not completely eliminate biases.

## 5. Conclusions

This study examined factors at the individual, household, and social group level associated with food insecurity in a quota-based sample of households residing in the 41 counties eligible for FEMA Individual Assistance following Hurricane Harvey. Older age, better physical health, better mental health, greater income, and a high level of social support were protective against food insecurity following Harvey. Minority (non-white) race, economic instability due to loss of income or a job related to the storm, major home damage due to Harvey, relocation as a result of Harvey, and receiving assistance from a community organization were risk factors for food insecurity. This study adds to the scant literature on food insecurity outcomes following disasters in the US. Characterizing risk and protective factors for post-disaster food insecurity contributes evidence to establishing consistency, strength of association, and specificity for identifying causal drivers of post-disaster food insecurity [37]. Future research examining change in food security status from pre-event to post-event, specifically longitudinal studies of the impact of disasters on disaster-affected households and the local food system, are important next steps in this research to better characterize the impact of the disaster of food security outcomes. Further studies should also focus not only on food security outcomes but changes in diet and eating patterns for displaced individuals to understand nutritional impacts beyond food insecurity that may result from changes in eating patterns during times of disruption. For disaster managers and public health officials, including food security in disaster planning and post-event rapid assessment can help to reduce food insecurity among affected households, especially those most vulnerable to adverse disaster consequences. Disaster management policies and programs for disaster-affected households that jointly address food security with housing security may reduce vulnerability for households experiencing housing losses or displacement. For example, connecting the Disaster—Supplemental Nutrition Assistance Program (D-SNAP) with FEMA Individual Assistance Programs might be one way to reduce the administrative burden of navigating two separate application processes and systems on disaster affected families while providing additional support during long-term recovery [100,101].

## Figures and Tables

**Table 1:** Characteristics of the sample.

Characteristics	Frequency	Percent
Food insecurity reported after Harvey	424	42.3
**Individual Level**		
*Age*		
18–44 years: Early working age	512	51.1
45–64 years: Later working age	337	33.6
65+ years: Retirement age	153	15.3
*Race*		
White	574	57.3
Black	101	10.1
Hispanic	275	27.5
Other	51	5.1
*Sex*		
Male	427	42.6
Female	575	57.4
*Employment status*		
Unemployed	106	10.6
*Education*		
High school	281	28.0
Some college	428	42.7
College degree	195	19.5
Graduate degree	98	9.8
*Physical Health*		
Well below the general population	173	17.3
Below the general population	112	11.2
Same or better than the general population	715	71.5
*Mental Health*		
Well below the general population	295	29.5
Below the general population	133	13.3
Same or better than the general population	572	57.2
**Household Level**		
*Income*		
Less than $15,000	133	14.2
$15,000–24,999	114	12.2
$25,000–34,999	103	11.0
$35,000–49,999	138	14.8
$50,000–74,999	187	20.0
$75,000–99,999	122	13.1
$100,000–149,999	78	8.4
$150,000 or more	59	6.3
*Economic instability*		
Job/income loss due to Harvey	528	52.7
*Homeownership*		
Home owner	625	62.4
*Home damage*		
Little to no damage	895	89.3
Major damage	107	10.7
*Relocation*		
Relocated due to Harvey	165	16.5
*Geography*		
Rural	487	48.6
**Social Level**		
*Social support*		
High social support	765	76.4
*Disaster assistance*		
Community-based organization assistance	90	9.0

**Table 2:** Frequency of individual, household, and social factors among the food secure and insecure.

Factor	Food Secure	Food Insecure
	*n* (within col %)	*n* (within col %)
Individual Level		
*Age* ***		
18–44 years: Early working age	232 (40.1)	280 (66.0)
45–64 years: Later working age	226 (39.1)	111 (26.2)
65 years +: Retirement age	120 (20.8)	33 (7.8)
*Race* ***		
White	391 (67.8)	183 (43.2)
Black	44 (7.6)	57 (13.4)
Hispanic	126 (21.8)	149 (35.1)
Other	16 (2.8)	35 (8.3)
*Sex*		
Male	243 (42.0)	184 (43.4)
Female	335 (58.0)	240 (56.6)
*Employment status* ***		
Unemployed	42 (7.3)	64 (15.1)
*Education* ***		
High school	133 (23.0)	148 (34.9)
Some college	238 (41.2)	190 (44.8)
College degree	139 (24.1)	56 (13.2)
Graduate degree	68 (11.8)	30 (7.1)
*Physical Health* ***		
Well below the general population	65 (11.3)	108 (25.6)
Below the general population	45 (7.8)	67 (15.9)
Same or better than the general population	468 (81.0)	247 (58.5)
*Mental Health* ***		
Well below the general population	108 (18.7)	187 (44.3)
Below the general population	64 (11.1)	69 (16.4)
Same or better than the general population	406 (70.2)	166 (39.3)
**Household Level**		
*Income* ***		
Less than $15,000	38 (7.1)	95 (23.7)
$15,000–24,999	50 (9.4)	64 (16.0)
$25,000–34,999	51 (9.6)	52 (13.0)
$35,000–49,999	78 (14.6)	60 (15.0)
$50,000–74,999	131 (24.6)	56 (14.0)
$75,000–99,999	78 (14.6)	44 (11.0)
$100,000–149,999	61 (11.4)	17 (4.2)
$150,000 or more	46 (8.6)	13 (3.2)
*Economic instability* ***		
Job/income loss due to Harvey	230 (39.8)	298 (70.3)
*Homeownership* ***		
Home owner	416 (72.0)	209 (49.3)
*Home damage* ***		
Little to no damage	554 (95.9)	341 (80.4)
Major damage	24 (4.2)	83 (19.6)
*Relocation* ***		
Relocated due to Harvey	55 (9.5)	110 (25.9)
*Geography*		
Rural	294 (50.9)	193 (45.5)
**Social Level**		
*Social support* ***		
High social support	500 (86.5)	265 (62.5)
*Disaster assistance* ***		
Community-based organization assistance	24 (4.2)	66 (15.6)

*** *p* < 0.001 of χ^2^ test

**Table 3:** Likelihood of food insecurity across individual, household, and social factors.

Factor	Model 1: Individual Level		Model 2: Household Level		Model 3: Social Level	
	OR	95% CI	OR	95% CI	OR	95% CI
*Age*						
18–44 years: Early working age	referent		referent		referent	
45–64 years: Later working age	**0.53**	**(0.37, 0.76)**	**0.59**	**(0.40, 0.87)**	**0.49**	**(0.32, 0.73)**
65+ years: Retirement age	**0.33**	**(0.19, 0.59)**	**0.43**	**(0.23, 0.79)**	**0.40**	**(0.22, 0.75)**
*Race*						
White	referent		referent		referent	
Black	**2.42**	**(1.48, 3.98)**	**1.75**	**(0.99, 3.11) ¹**	**1.79**	**(1.01, 3.18)**
Hispanic	**1.80**	**(1.24, 2.61)**	**1.68**	**(1.11, 2.55)**	**1.67**	**(1.09, 2.54)**
Other	**3.85**	**(1.91, 7.75)**	**4.78**	**(2.17, 10.51)**	**4.39**	**(1.96, 9.82)**
*Employment status*						
Unemployed	1.22	(0.77, 1.95)				
*Education*						
High school	referent					
Some college	0.94	(0.66, 1.32)				
College degree	**0.60**	**(0.39, 0.94)**				
Graduate degree	0.83	(0.47, 1.47)				
*Physical Health*						
Well below the general population	referent		referent		referent	
Below the general population	0.86	(0.50, 1.48)	0.98	(0.54, 1.79)	1.09	(0.59, 2.02)
Same or better than the general population	**0.31**	**(0.21, 0.46)**	**0.42**	**(0.27, 0.64)**	**0.46**	**(0.29, 0.71)**
*Mental Health*						
Well below the general population	referent		referent		referent	
Below the general population	**0.63**	**(0.40, 1.00) ¹**	**0.59**	**(0.35, 0.98)**	0.61	(0.36, 1.03)
Same or better than the general population	**0.29**	**(0.21, 0.40)**	**0.37**	**(0.26, 0.53)**	**0.46**	**(0.32, 0.67)**
*Income*						
Less than $15,000			referent		referent	
$15,000–24,999			**0.53**	**(0.29, 0.98)**	0.60	(0.32, 1.11)
$25,000–34,999			**0.50**	**(0.27, 0.94)**	0.55	(0.29, 1.04)
$35,000–49,999			**0.47**	**(0.26, 0.85)**	0.57	(0.31, 1.04)
$50,000–74,999			**0.25**	**(0.14, 0.43)**	**0.29**	**(0.16, 0.51)**
$75,000–99,999			**0.44**	**(0.24, 0.81)**	0.54	(0.29, 1.00)
$100,000–149,999			**0.24**	**(0.11, 0.52)**	**0.27**	**(0.13, 0.57)**
$150,000 or more			**0.20**	**(0.09, 0.47)**	**0.23**	**(0.10, 0.53)**
*Economic instability*						
Job/income loss due to Harvey			**2.42**	**(1.74, 3.38)**	**2.43**	**(1.73, 3.41)**
*Homeownership*						
Homeowner			0.75	(0.53, 1.06)		
*Home damage*						
Major home damage			**2.41**	**(1.28, 4.55)**	**2.11**	**(1.12, 3.98)**
*Relocation*						
Relocated due to Harvey			**1.86**	**(1.15, 3.02)**	**1.89**	**(1.15, 3.09)**
*Social support*						
High social support					**0.37**	**(0.25, 0.55)**
*Disaster assistance*						
Community-based organization assistance					**1.99**	**(1.11, 3.58)**
Log Likelihood	−558.02		−469.97		−456.89	
Akaike Information Criterion	1146.04		983.94		959.79	
Bayesian Information Criterion	1219.64		1090.34		1071.02	

Note: Bolded figures are statistically significant at the *p* ≤ 0.05 level.
